# Identification, Molecular Characteristic, and Expression Analysis of PIFs Related to Chlorophyll Metabolism in Tea Plant (*Camellia sinensis*)

**DOI:** 10.3390/ijms222010949

**Published:** 2021-10-11

**Authors:** Xiangna Zhang, Ligui Xiong, Yong Luo, Beibei Wen, Kunbo Wang, Zhonghua Liu, Jian-an Huang, Juan Li

**Affiliations:** 1 Key Laboratory of Tea Science of Ministry of Education, Hunan Agricultural University, Changsha 410128, China; xynzzxn529@163.com (X.Z.); xiongligui@hunau.edu.cn (L.X.); jdllqcclfc@163.com (B.W.); wkboo163@163.com (K.W.); larkin-liu@163.com (Z.L.); 2 National Research Center of Engineering Technology for Utilization of Functional Ingredients from Botanicals, Co-Innovation Centre of Utilization of Functional Ingredients from Botanicals, Hunan Agricultural University, Changsha 410128, China; 3 School of Chemistry and Environmental Science, Xiangnan University, Chenzhou 423043, China; luoyong@xnu.edu.cn

**Keywords:** *Camellia sinensis*, phytochrome-interacting factors, transcription factors, chlorophyll, gene expression

## Abstract

The phytochrome-interacting factors (PIFs) proteins belong to the subfamily of basic helix–loop–helix (bHLH) transcription factors and play important roles in chloroplast development and chlorophyll biosynthesis. Currently, knowledge about the *PIF* gene family in *Camellia sinensis* remains very limited. In this study, seven PIF members were identified in the *C. sinensis* genome and named based on homology with *AtPIF* genes in *Arabidopsis thaliana.* All *C. sinensis* PIF (CsPIF) proteins have both the conserved active PHYB binding (APB) and bHLH domains. Phylogenetic analysis revealed that CsPIFs were clustered into four groups—PIF1, PIF3, PIF7, and PIF8—and most CsPIFs were clustered in pairs with their corresponding orthologs in *Populus tremula*. *CsPIF* members in the same group tended to display uniform or similar exon–intron distribution patterns and motif compositions. *CsPIF* genes were differentially expressed in *C. sinensis* with various leaf colors and strongly correlated with the expression of genes involved in the chlorophyll metabolism pathway. Promoter analysis of structural genes related to chlorophyll metabolism found DNA-binding sites of PIFs were abundant in the promoter regions. Protein–protein interaction networks of CsPIFs demonstrated a close association with phytochrome, PIF4, HY5, TOC1, COP1, and PTAC12 proteins. Additionally, subcellular localization and transcriptional activity analysis suggested that CsPIF3b was nuclear localized protein and possessed transcriptional activity. We also found that CsPIF3b could activate the transcription of *CsHEMA* and *CsPOR* in *Nicotiana benthamiana* leaves. This work provides comprehensive research of CsPIFs and would be helpful to further promote the regulation mechanism of PIF on chlorophyll metabolism in *C. sinensis*.

## 1. Introduction

Tea plant (*Camellia sinensis* (L.) O. Kuntze) is an important perennial woody plant, rich in active ingredients beneficial to human health, and has special drinking and economic value. Leaf color is an important trait that can be easily identified in the breeding process of plants. With the deciphering of the whole genome of tea plants and the development of modern biological technology, various tea cultivars exhibiting leaf color variation have been developed [1,2,3]. In particularly, high-theanine-content tea cultivars with different leaf colors are attracting increasing attention due to the unique color and multiple health benefits. According to the color of leaves, high-theanine tea cultivars can be divided into three types: green, white and yellow [4]. Numerous studies have demonstrated that tea cultivars with white or yellow leaves are mainly caused by the decrease of chlorophyll content, which is related to the abnormal chloroplast development, including changes of chloroplast structure, decreased the number of chloroplasts and granular layer, disintegration of the matrix lamella [5,6].

Chlorophyll is a key pigment that presents in plant chloroplasts and participates in photosynthesis, which plays essential role in photosynthetic light-harvesting and transferring electrons to the photoreaction center [7]. In addition, chlorophyll regulates the expression of photosynthesis-related genes through plastid signals and maintains the stability of proteins in chloroplasts [8,9,10]. In tea plant, chlorophyll corresponds to the green pigments in leaves and forms the color of leaves together with carotenoids, which also plays an extremely important role in growth and yield [11]. It has been demonstrated that the formation of chlorophyll a took part in the regulation of the catechins composition in tea plants [12]. To a certain extent, the composition and content of chlorophyll affect the quality of tea. Previous studies have revealed that the chlorophyll biosynthesis pathway involves the formation of 5-aminolevulinic acid (ALA), biosynthesis of protoporphyrin IX, biosynthesis of chlorophyll a and b, and degradation of chlorophyll [13]. Throughout this process, genes that encode key enzymes for regulating chlorophyll accumulation are well documented [14]. However, research on these genes in tea plants remains limited and the underlying transcriptional regulation of these genes is less well understood.

Multiple phytochrome-interacting factors (PIFs) family proteins (PIF1, PIF3, PIF4, PIF5) were found to be involved in chloroplast development and affected chlorophyll content by regulating the expression of genes related to chlorophyll biosynthesis or degradation in *Arabidopsis thaliana* [15]. For example, AtPIF3 has been shown to regulate chlorophyll biosynthesis by directly binding to *HEMA1*, *GUN5* [16]. It is reported that PIF1 regulated the expression of *PORC*, *FeChII*, and *HO-3* involved in controlling chlorophyll biosynthesis in the dark, and light-induced degradation of PIF1 could relieve this negative regulation to promote photomorphogenesis in *A.* *thaliana* [17]. Moreover, AtPIF1 activated transcription of *PORC* by directly binding to a G-box (CACGTG) DNA sequence element presented in its promoter. Overexpression of PIF4 and PIF5 significantly induced leaf senescence through chlorophyll degradation in darkness, as well as the activity levels of *SGR*, *NYC1*, *NOL*, and *PAO* [18,19]. In addition, PIF could interact with other proteins to regulate chlorophyll biosynthesis. It was found that PIF1 interacts with EIN3/EIL1 protein for inhibiting the accumulation of Pchlide, so pif1 mutant is prone to photobleaching. PIF1 also interacts with the DNA-binding domain of FHY3 to partially inhibit the expression of *HEMB1*, therefore affecting the precursor of chlorophyll biosynthesis [20]. PIF3 interacts with HY5 to regulate BBX11, therefore suppressing the level of protochlorophyllide in the dark and promoting photomorphogenesis under light [21]. Together, these studies indicated the extensive roles of PIFs in regulating chlorophyll biosynthesis. Despite the critical importance of PIFs involved in light signaling pathway has been documented in other plants, the role of PIF has not been systematically characterized in tea plant, especially identification, expression pattern, and molecular characteristics.

In this study, we identified seven members of the PIF family in *C. sinensis* and performed systematic analysis, including their amino acid sequence, phylogeny, gene structure, and motif compositions. We also analyzed the expression levels of seven *CsPIF* genes in tea plants with various leaf colors, and explored the relationship between them and the expression of genes related to chlorophyll metabolism. In addition, the molecular characteristic and function of CsPIF3b were characterized. These results lay a foundation for understanding of the CsPIFs related to the chlorophyll metabolism and could further promote studies on their potential functions in tea plants.

## 2. Results

### 2.1. Identification of CsPIF Family Members in Tea Plants

A total of seven members (1 CsPIF1, 2 CsPIF3, 2 CsPIF7 and 2 CsPIF8) were confirmed as CsPIF proteins and named according to homology with AtPIFs (Table 1). The CDS and protein sequences of CsPIF members were provided in Table S1. By analyzing the physical and chemical properties analysis of seven CsPIFs, *CsPIF8a* encodes the smallest protein (416 amino acids), and *CsPIF3a* encodes the largest protein (723 amino acids). The molecular weight of proteins ranges from 44.84 kDa (CsPIF8a) to 77.34 kDa (CsPIF3a), and their isoelectric points are between 5.88 (CsPIF3a) and 9.26 (CsPIF7b). The instability index of PIF members in tea plants was greater than 40, indicating that PIF members in tea plants might be unstable. The Grand average of hydropathicity (GRAVY) were all negative, indicating that all CsPIF proteins are hydrophilic.

### 2.2. Amino Acid Sequence and Phylogenetic Analysis of CsPIF Proteins

**Figure 1:**
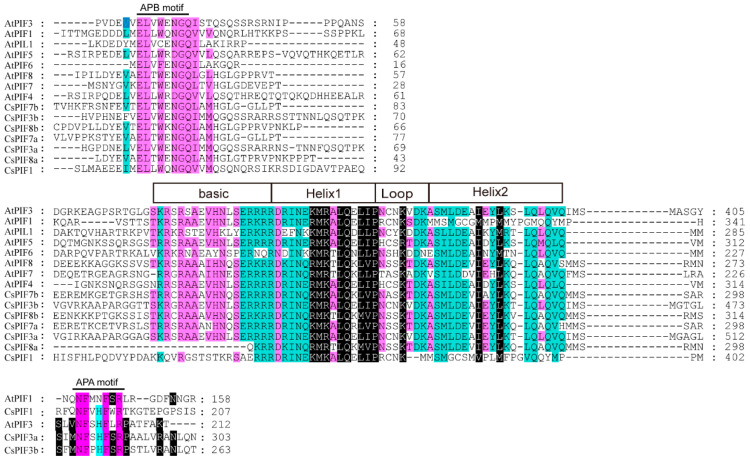
Multiple sequence alignment of PIF proteins in *C. sinensis* and *A. thaliana*. Conserved domain is divided into three subdomains (APB, HLH and APA), which are marked with lines above the sequences.

**Figure 2:**
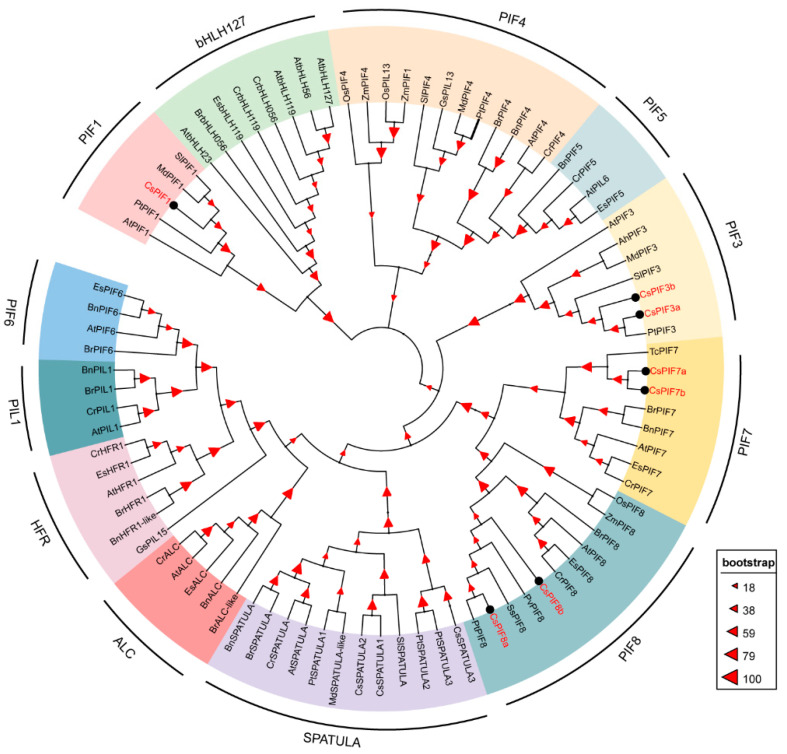
Phylogenetic tree of PIF proteins in tea plant and other plants. Phylogenetic analysis of PIF protein in *C. sinensis* performed with 86 sequences from 13 other plant species. All the sequences of PIF proteins are detailed in Table S1. PIF proteins sequences were aligned by ClustalX 1.83, and the phylogenetic tree was constructed using MEGA 7.0 by the neighbor-joining (NJ) method and visualized using the online software iTOL. Each of clusters is colored differently, and the seven CsPIFs are colored red.

Amino acid sequence alignment analysis of CsPIF proteins showed that all the CsPIF members contained both the bHLH and APB domains, only CsPIF1, CsPIF3a, and CsPIF3b contained APA domain (Figure 1). To analyze the evolutionary relationships of PIF in tea plant and other plants, an unrooted phylogenetic tree was constructed using full-length amino acid sequences (Figure 2, Supplementary Materials Text S1). Based on the resulting phylogenetic tree, seven CsPIF proteins were divided into PIF1, PIF3, PIF7, and PIF8 clades. Within each clade, most CsPIFs were clustered in pairs with their corresponding orthologs in *Populus tremula*. For instance, PIF1, PIF3, and PIF8 in tea plant were clustered with their homologous sequences in *P. tremula* (PtPIF). CsPIF7a and CsPIF7b showed close relationships with *Theobroma cacao* (TcPIF7), and similarity in their amino acid sequences was 64.84% (Figure S1).

### 2.3. Gene Structural Analysis and Motif Analysis of CsPIFs

**Figure 3:**
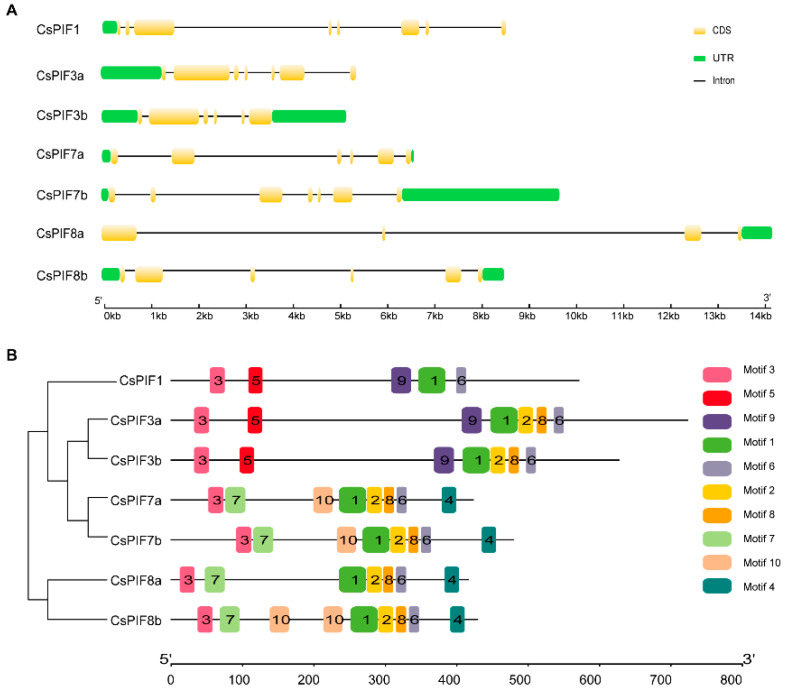
Gene structure and motif composition in CsPIF family. (**A**) The diagram was drawn using the online Gene Structure Display Server (GSDS 2.0) (http://gsds.gao-lab.org (accessed on 8 July 2021)) with full-length gene sequence and CDS sequence of *CsPIF* genes. CDS and UTR are indicated by yellow and green boxes respectively, and introns are represented by gray lines. (**B**) The motifs, numbers 1–10, are represented by different colored boxes. The information of each motif is provided in Table S3.

The number and location of intron and exon in *CsPIF* gene were predicted by GSDS on the CDS and full-length sequences of *CsPIF* genes. As shown in Figure 3A, all genes had intron and exon structures, and the number of exons varies from 4 to 8. The CsPIF8a gene had the least number of exons and introns (4 and 3, respectively). In addition, PIF members in the same clade shared the same number of exons and introns, although their localization was different.

To further investigate the sequence features of CsPIF proteins, 10 conserved motifs of PIF members named Motif1-Motif10 respectively were identified by the MEME website (Figure 3B). The lengths of these conserved motifs vary from 15 to 39 amino acids, and details of motifs were outlined in Table S2. Each gene branch had similar motif composition, which confirmed the reliability of phylogenetic tree analysis. The CsPIF members contained 5–9 conserved motifs, and PIF1 had the least number of motifs. Motif 3 (APB domain) and Motif 6 were present in all CsPIF proteins. Motif 5 (APA domain) was exclusively identified in CsPIF1, CsPIF3a, and CsPIF3b. Motif 7 was found in the more evolutionarily related CsPIF7a, CsPIF7b, and CsPIF8a, CsPIF8b proteins. Additionally, although PIF8a and PIF8b were clustered into the same clade, their motif composition differed.

### 2.4. Expression Patterns and Correlation Analysis of CsPIFs and Structural Genes Related Chlorophyll Metabolism in Different Leaf Color Tea Cultivars

**Figure 4:**
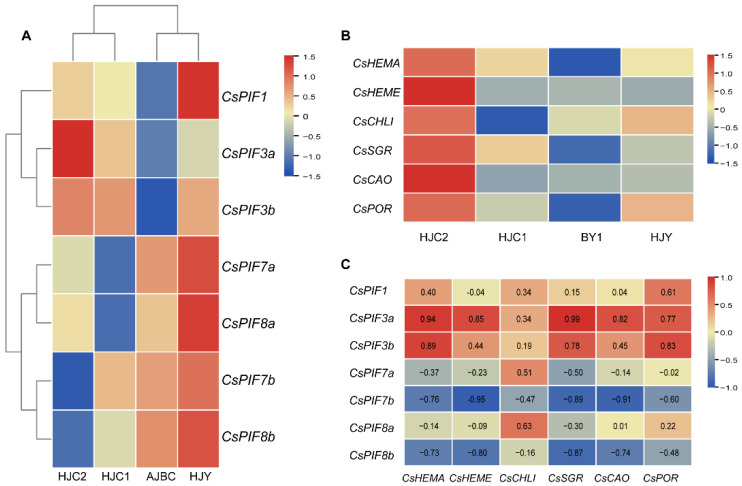
Expression patterns and correlation analysis of CsPIFs and structural genes related chlorophyll metabolism. (**A**) Expression patterns of CsPIFs in tea leaves of different leaf color tea cultivars. (**B**) Expression patterns of structural genes related chlorophyll metabolism in leaves of different leaf color tea cultivars. The red-blue schemes are labelled on the right side of heat map, and red to blue represent high to low expression levels. (**C**) Correlation between CsPIFs and structural genes related chlorophyll metabolism.

Expression patterns of *CsPIF* genes and structural genes related to chlorophyll metabolism in tea cultivars with different leaf colors were performed by qRT-PCR. The expression patterns of *CsPIF* genes varied substantially in different leaf color tea cultivars. Cluster analysis showed *CsPIFs* clustered into two categories according to the expression difference between green-leaf and albino tea cultivars (Figure 4A). CsPIF7a, CsPIF8a, and CsPIF7b, CsPIF8b were significantly down-regulated in HJC1 and HJC2, respectively. Conversely, the remaining three *PIFs* (*CsPIF1*, *CsPIF3a*, *CsPIF3b*) were significantly lower expressed in BY1 or HJY. Especially the expression pattern of *CsPIF3a* was almost consistent with the green degree of leaves. Six structural genes (*HEMA*, *HEME*, *CHLI*, *POR*, *CAO* and *SGR*) related to chlorophyll metabolism were expressed at highest levels in HJC2 than the other three tea cultivars, whereas most of them showed the lowest expression in BY1 (Figure 4B). It was noteworthy that the expression of *CsCHLI* and *CsPOR* genes, which are important genes in the process of chlorophyll biosynthesis, were slightly up-regulated in the albino tea cultivar HJY, suggesting that chlorophyll synthesis might be active in HJY. Moreover, the correlation analysis between the expression of *CsPIFs* and structural genes was conducted (Figure 4C). The result showed that the expression levels of *CsPIFs* were significantly positively (*CsPIF3a*, *CsPIF3b*) or negatively (*CsPIF7b*, *CsPIF8b*) correlated with the expression of structural genes related to chlorophyll metabolism.

### 2.5. Protein–Protein Interaction Network of CsPIFs and Binding Elements in Promoters of Chlorophyll Metabolism-Related Gene

**Figure 5:**
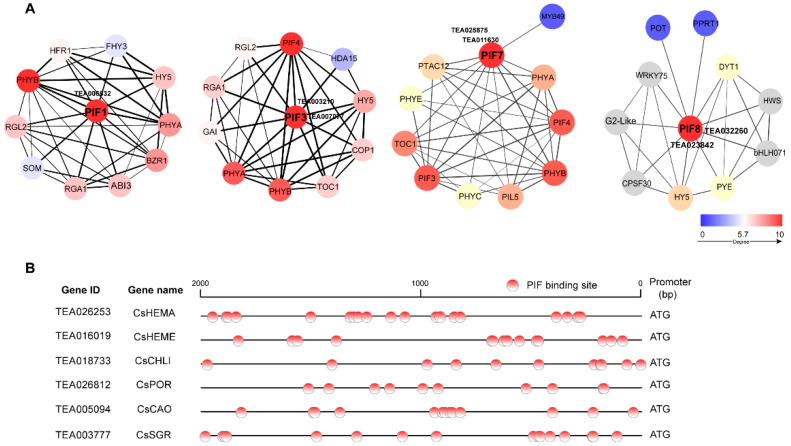
Analysis of protein–protein interaction networks and binding sites in promoters of CsPIF transcription factors. (**A**) Interaction networks of CsPIFs with other proteins. The thickness of the edge represents the strength of the association between two nodes. The intensity of protein interaction was represented by the color gradient, and blue to red represent low to high degree between the node and hub proteins. (**B**) DNA-binding sites of CsPIF proteins in promoter regions of structural genes in the chlorophyll metabolism pathway. The possible DNA-binding sites are marked with red point.

To gain insight into the regulatory function of CsPIF proteins, protein–protein interaction networks were predicted based on the interactions of homologous proteins in *A. thaliana*. CsPIF proteins were successfully mapped to AtPIF1, AtPIF3, AtPIF7, and AtPIF8, respectively. The degree correlations of interaction with the hub-protein in the protein–protein interaction networks were expressed by the color gradient. Among them, PIF1, PIF3, and PIF7 were highly connected hub proteins and could interact with phytochrome, PIF4, HY5, TOC1, COP1 and PTAC12 proteins (Figure 5A), which are involved in the regulation of photomorphogenesis, light regulation, nitrogen metabolism, and chloroplast development in plants. In addition, CsPIF1 and CsPIF3 also interact with GAI/RGA proteins of DELLA family, which are negative regulators of GA signal transduction. Besides HY5, CsPIF8 also interacts with CPSF30, K+ transporter POT, and PPRT, which are involved in ABA hormone signal transduction, transport of K+ and nitrogen assimilation.

CsPIFs belong to the bHLH transcription factor family subfamily, with the ability to specifically recognize and bind G-box and E-box. To further explore the potential regulatory roles of CsPIFs in chlorophyll metabolism, the upstream promoter region of the differential structural genes in the chlorophyll metabolic pathway was analyzed using JASPAR2020 database (http://jaspar.genereg.net (accessed on 1 September 2021)). As shown in Figure 5B and Text S2, PIF-binding sites were abundant in the promoter regions of structural genes in the chlorophyll metabolic pathway, suggesting that CsPIFs might participate in the chlorophyll metabolism by regulating the expression level of structural genes. 

### 2.6. Subcellular Localization and Transcriptional Activation of CsPIF3b

**Figure 6:**
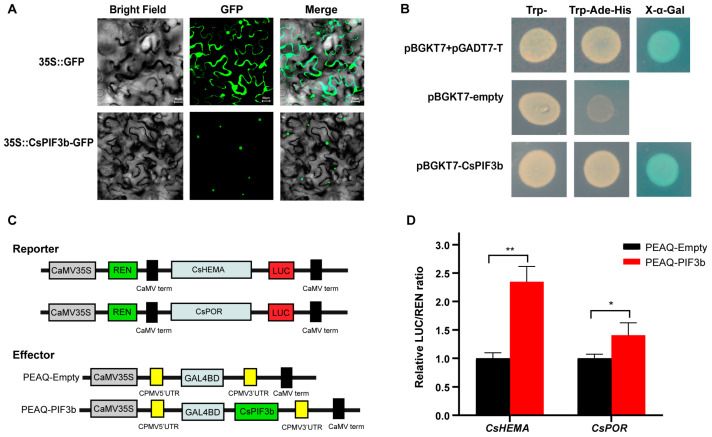
Molecular Characteristics of CsPIF3b protein. (**A**) Subcellular localization of CsPIF3b in N. benthamiana leaves. Images with GFP signals were taken using fluorescent microscope. Bars, 50 μm. (**B**) Transcriptional activation activity of CsPIF3b in yeast cells. The transcriptional activation ability of CsPIF3b were evaluated based on growth and X-α-Gal activity of yeast on SD plates (SD/-Trp/-His/-Ade). (**C**) Diagrams of the reporter and effector constructs used in dual-luciferase transient expression system. (**D**) Trans-activation of CsPIF3b to promoter of chlorophyll-related genes by CsPIF3b in N. benthamiana leaves. The trans-activation of CsPIF3b to *CsHEMA* and *CsPOR* were determined by the LUC/REN ratio. Bar graphs are expressed as mean ± SEM, * *P* < 0.05; ** *P* < 0.01 vs. the PEAQ-Empty group.

As transcriptional regulators, PIFs have been reported to be nuclear proteins. To examine the subcellular localization, GFP vector and recombinant plasmids (CsPIF3b-GFP) were transformed into *Nicotiana benthamiana* leaves. As shown in Figure 6A, GFP signals of CsPIF3b-GFP fusion protein were only observed in the nucleus, whereas GFP protein was distributed throughout the cell, suggesting that CsPIF3b localized in the nucleus, consistent with potential function as a transcription regulator.

The transcriptional activity of CsPIF3b was analyzed using a GAL4-responsive reporter system in yeast. Yeast cells transformants transformed with negative control (pGBKT7 vector) did not grow in the SD/-Trp/-His/-Ade medium and showed no X-α-Gal activity. However, cells transformed with pGBKT7-CsPIF3b and the positive control (pGBKT7–p53+pGADT7-T) grew well and showed X-α-Gal activity in the SD/-Trp/-His/-Ade medium (Figure 6B). The result indicated that CsPIF3b might act as a transcriptional activator with activation activity.

### 2.7. CsPIF3b Positively Regulate the Expression of CsHEMA and CsPOR

To determine on whether the CsPIF3b could regulate the promoters of *CsHEMA*, *CsPOR*, we performed transient dual-luciferase reporter assays in *N. benthamiana* leaves using CsHEMA pro-LUC and CsPOR pro-LUC as reporters and recombinant plasmid CsPIF3b-pEAQ as the effector (Figure 6C). As shown in Figure 6D, the LUC/REN ratio was significantly decreased when CsPIF3b was co-transfected with CsHEMA pro-LUC and CsPOR pro-LUC, compared with the co-transfection of the reporters with the empty vector. These results suggested that CsPIF3b could activate the expression of chlorophyll biosynthesis related genes *CsHEMA* and *CsPOR* to positively regulate the chlorophyll accumulation.

## 3. Discussion

PIFs, as a subfamily of the bHLH transcription factor family, play key regulatory roles in plant growth and development. With the availabilities of the whole genome sequence of many plants, PIF families in several species have been identified, such as *A*. *thaliana*, *Oryza sativa*, *P. tremula*, *Brassica napus*, *Solanum lycopersicum* [22,23,24,25,26]. However, little is known about PIF family in tea plants. Here, we identified CsPIF members and explored their regulatory function on chlorophyll biosynthesis in tea plants.

### 3.1. Identification and Characterization of PIF Genes in Tea Plants

Based on the eight AtPIF protein sequences, we identified seven PIF members from the *C. sinensis* genome and split them into four groups: PIF1, PIF3, PIF7, PIF8 (Table 1, Figure 1). Members of the PIF4, PIF5, and PIF6 were not identified in *C. sinensis* genome, probably due to gene loss during plant evolution. The number of *PIF* gene members in *C. sinensis* was comparable to that of other diploid plant species, such as eight *PIF* genes in *A. thaliana*, six in *O. sativa*, seven in *S. lycopersicum,* presumably resulting from the evolutionary conservation of the gene family.

PIF proteins have been reported to contain APB and APA domains in addition to bHLH conserved domains. The importance of the APA and APB motifs is to provide Pfr specificity for the physical interaction between PIF and phytochrome A (phyA) and phytochrome B (phyB), respectively. In *A. thaliana*, eight PIFs have APB domain, only PIF1 and PIF3 have both APB and APA domain [27]. In *Zea mays*, the analysis of amino acids sequences illustrates the presence of seven ZmPIFs with a conserved APB motif, of which three (ZmPIF3.1, ZmPIF3.2 and ZmPIF3.3) also contain the APA motif [28]. Among six *O. sativa* PILs, all proteins (OsPIL11-OsPIL16) have the conserved APB domain; however, only OsPIL15 contains an APA domain [24]. Similarity, in this study, all the CsPIF members contained both the bHLH and APB domains, only CsPIF1, CsPIF3a, and CsPIF3b contained APA domain (Figure 1), suggesting that they probably physically interact with phyA and phyB. Moreover, phylogenetic analysis of PIFs in *C. sinensis* and other plants showed that CsPIFs were highly orthologous to corresponding *PIFs* genes in *P. tremula* or *T. cacao* (Figure 2), indicating that *PIF* genes differentiate between woody and herbaceous plants. The number of exons and introns of the CsPIF1, CsPIF3, and CsPIF7 gene was similar to that of *A. thaliana*, *S. lycopersicum* and *Malus domestica*, implying that *PIF* genes might perform similar functions in different plant species. *CsPIF* genes from the same group shared the same number of exons and introns (Figure 3A), except that *CsPIF8a* contained the smallest number of exons and introns (4 and 3 respectively), indicating that the *PIF8* gene might be functionally differentiated in tea plants. In addition, it has been shown that PIFs can bind to cis elements of downstream genes through their basic region of bHLH domain [29]. Although the domain was highly conserved, several amino acid sites were missing in CsPIF8a protein (Figure 1). The sequence variation within bHLH domain suggested that CsPIF8a may have different protein–DNA binding specificities and physiological roles in *C. sinensis.*

### 3.2. CsPIF Correlated with the Expression of Chlorophyll Metabolism Genes

There is considerable evidence that *PIF is* potentially involved in regulating the expression of chlorophyll and photosynthetic genes in *A. thaliana* [16,18,30], *Z. may* [31] and *O. sativa* [32]. However, little and fragmented information is currently available about the expression of PIF genes at different chlorophyll levels. To explore the functional of *CsPIF* genes in the modulation of the chlorophyll, we investigated the expression of *CsPIF* genes at different chlorophyll levels in tea plants. The *CsPIFs* displayed differential mRNA accumulation pattern. The transcript levels of two homoeologous pairs of *CsPIF3* (*CsPIF3a*/*CsPIF3b*) were significantly down-regulated in BY1 with low chlorophyll content (Figure 4A). Similar in *O. sativa,* the knockdown mutation in OsPIL1 resulted in the pale-green leaf phenotype [32]. Additionally, the expression level of *SlPIF4* increased during deetiolation. On the contrary, the expression of CsPIF7 and CsPIF8 was significantly up-regulated in BY1, indicating that *CsPIFs* had undergone functional specification. Many types of research have revealed the importance of key genes involved in the chlorophyll metabolic pathway in plants [33,34]. *HEMA*, *HEME*, *CHLI*, *POR*, *CAO*, and *SGR* are important genes in each stage of chlorophyll synthesis, which are related to chlorophyll content [3]. Previous studies in *C. camellia* had shown that *CHLG* and *CAO* were significantly expressed higher in the green stage than that in the albino stage of “BY1” [35]. Additionally, the down-regulation of *POR* gene under strong light results in the decrease of chlorophyll content and the appearance of the etiolated phenotype of “Baijiguan” [36]. In this study, we compared the expression patterns of six key genes in chlorophyll metabolic pathway and observed substantial differences. Almost all the six genes showed a high expression level in HJC2 and low in BY1, which was consistent with the chlorophyll level (Figure 4B). However, the expression of the *SGR* gene involved in chlorophyll degradation was the highest in the dark-green cultivar HJC2. It could be deduced that the function of *SGR* to degrade chlorophyll mainly occurred in the fruit ripening period or the leaf senescence period, rather than the tender stage. In addition, the correlation analysis between the expression of *CsPIFs* and structural genes showed that *CsPIF3a* and *CsPIF3b* were significantly positively correlated with structural genes, while *CsPIF7b* and *CsPIF8b* were negatively correlated with them. Therefore, these CsPIF transcription factors could be considered candidates in the regulation of the accumulation of chlorophyll in tea plants.

### 3.3. CsPIF Involved in the Transcriptional Regulation of Chlorophyll Metabolism

PIFs have been shown to perform regulatory functions by interacting with many other cellular signaling molecules. For example, AtPIF3 was found to directly interact with phytochrome to regulate the expression of downstream genes of PHYA/B signal [37]. In addition, AtPIF4 and AtPIF5 were shown to form heterodimers with HFR1 to regulate the protein level in shade-avoidance reaction [38]. DELLA proteins were predicted to interact with PIF3 and PIF4 to inhibit their activity and promote photomorphogenesis. Therefore, PIFs can be considered to be important candidates for regulating plant growth [39]. The protein–protein interaction analyses of CsPIF TFs revealed that these proteins interacted closely with PHY, HFR, HY5, COP1, TOC1 and DELLA proteins (Figure 5A), similar to previous studies. PIFs belong to the 15th subfamily of bHLH and preferentially bind a G-box (CACGTG) DNA sequence element, which is a subclass of an E-box element (CANNTG) present in many light-regulated promoters. It is widely documented that AtPIF1 and AtPIF3 regulated chlorophyll biosynthesis by binding G-box in the promoter region of structural genes. The rich DNA-binding sites of PIFs were observed in the promoters of structural genes (Figure 5B), indicating that CsPIFs play putative regulatory roles in chlorophyll metabolism. PIF proteins are reported to be typical nuclear proteins [31,40]. Similarly, this study showed that PIF3b was a nucleus-location protein (Figure 6A). Moreover, it has been well documented that the C-terminal dimerization domain of PIF TFs is responsible for transcriptional regulatory activity and DNA binding [41]. The PIFs can act either as transcriptional activators or repressors. Transcriptional activity analysis in yeast cells suggested that CsPIF3b exhibited the ability of transcriptional activation (Figure 6B). It has been well documented that PIFs modulated the chlorophyll biosynthetic pathway by binding to the promoter regions of genes. Furthermore, correlation analysis showed that CsPIF3b is highly correlated with the expression of chlorophyll biosynthesis genes. In this study, dual-luciferase analysis showed that CsPIF3b strongly activated the expression of *CsHEMA* and *CsPOR* (Figure 6C). This evidence demonstrated that CsPIF3b participated in the regulation of chlorophyll biosynthesis in tea plants, which extends our knowledge of the PIFs family in tea plants and how they participate in the regulation of chlorophyll metabolism.

## 4. Materials and Methods

### 4.1. Plant Materials

**Figure 7:**
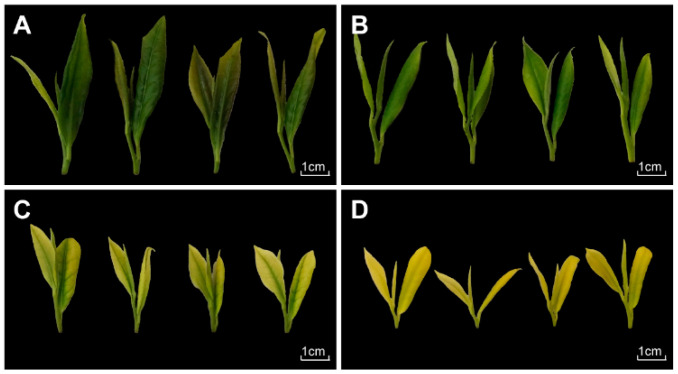
Leaf phenotypes of four tea cultivars used in this study. (**A**) ‘Huangjincha2’ (HJC2); (**B**) ‘Baojing Huangjincha1’ (BJ1); (**C**) ‘Baiye1’ (BY1); (**D**) ‘Huangjinya’ (HJY). Tea leaves were collected based on the “one bud and two leaves” standard. Bars, 1 cm.

Tea cultivar ‘Huangjincha2’ (HJC2), ‘Baojing Huangjincha1’ (BJ1), ‘Baiye1’ (BY1) and ‘Huangjinya’ (HJY) grown in the tea garden of the Hunan Tea Research Institute (latitude: 28.48° N, longitude: 113.36° E, Changsha, China) were used in this study. The color of HJC2 leaves showed dark-green and slightly purple and the color of BJ1 leaves showed light green; The color of BY1 leaves displayed milk-white and the color of HJY leaves exhibited yellow (Figure 7). One bud and two leaves were selected for the research, which were harvested and quickly frozen into liquid nitrogen and kept at −80 °C.

### 4.2. Identification of PIFs Transcriptional Factors

Bioedit software (V7.0.9.0) was used to establish a local database based on the tea plant genome [42]. Eight AtPIF proteins from TAIR (https://www.arabidopsis.org/ (accessed on 15 June 2021)) were used as query sequences to search the CsPIF proteins using BLAST search (http://tpdb.shengxin.ren (accessed on 15 June 2021)). Furthermore, conservative domain database (CDD) (https://www.ncbi.nlm.nih.gov/CDD (accessed on 15 June 2021)) was used for further determining whether CsPIF protein sequences contain the typical bHLH_AtPIF_like domain (cd11445) structure. Finally, the physiological and biochemical properties of CsPIF proteins for analyzed using ExPASy ProParam tool (https://web.expasy.org/protparam/(accessed on 22 July 2021)), and subcellular localization was predicted using Cell-PLoc 2.0 (http://www.csbio.sjtu.edu.cn/bioinf/Cell-PLoc-2/ (accessed on 22 July 2021)).

### 4.3. Sequence Alignments and Phylogenetic Analysis

The reference sequences of *Arabidopsis thaliana* and other plants were downloaded from the TAIR and NCBI websites, and multiple sequence alignments of CsPIFs and AtPIFs were performed using ClustalX 1.83 program (European Bioinformatics Institute, Cambridge, U.K.) [43] with default parameters. To survey the phylogenetic relationships of PIF genes in tea plant, PIF proteins in tea plant and other plants were used to construct a phylogenetic tree by the MEGA 7 with the neighbor-joining (NJ) method (p-distance model and 1000 bootstrap replicates). The phylogenetic tree was visualized in iTOL (https://itol.embl.de/login.cgi?logout=1# (accessed on 23 July 2021)) [44].

### 4.4. Gene Structure and Conserved Motifs Analysis

Gene CDS and exon structures of the CsPIF genes were predicted using the Gene Structure Display Server (GSDS2.0) web server (http://gsds.gao-lab.org/index.php (accessed on 25 July 2021)) with CDS and full-length gene sequences. Conserved motifs were identified using the Multiple Em for Motif Elicitation (MEME) (version 5.3.3) (https://meme-suite.org/meme/tools/meme (accessed on 26 July 2021)) with the motif width set to 6–50 and the maximum number of motifs set to 10. The Figures of gene structure and conserved motifs were generated using the TBtools (Toolbox for Biologists) software (v1.0986, Chengjie Chen, Guangzhou, China) [45].

### 4.5. qRT-PCR and Correlation Analysis of CsPIFs and Structural Genes Related Chlorophyll Metabolism

Total RNA was isolated from tea leaves using RNAprep Pure Plant Kit (Tiangen, Beijing, China) and 1 μg total RNA was reversed using the PrimeScript™ RT reagent Kit with gDNA Eraser (Takara, Dalian, China). Primers are listed in Table S1, and β-actin was used as an internal reference gene. qRT-PCR was performed on an ABI Quant Studio 3.0 PCR system (Applied Biosystems, Foster City, CA, USA) using TB Green™ Premix Ex Taq™ II (TaKaRa, Dalian, China). Triplicates of each experiment were performed for each gene expression test. The relative expression levels of selected genes were calculated using the 2^−ΔΔCt^ method. The heat maps were drawn using TBtools software (v1.0986) [45].

### 4.6. Analysis of the Protein–Protein Interaction (PPI) Network of PIF TFs and Promoter Regions of Structural Genes Related to Chlorophyll Metabolism

To investigate the transcriptional regulation of PIFs, the protein interaction networks of CsPIFs were constructed using a String database (V11.5) (https://string-db.org (accessed on 28 July 2021)). Additionally, the protein–protein interaction networks and the core protein in network were visualized using Cytoscape software (V3.8.2; Cytoscape Consortium, California, USA). The promoter sequence (upstream 2000 bp) of genes related to chlorophyll metabolism was downloaded from the tea plant genome. The PIFs binding sites on promoters were predicted using JASPAR^2020^ (http://jaspar.genereg.net (accessed on 1 September 2021)).

### 4.7. Subcellular Localization and Transcriptional Activation Assays of PIFs

Open reading frame sequence of CsPIF3b was amplified without stop codons via reverse transcription PCR using PrimeSTAR^®^ Max DNA polymerase (Takara, Dalian, China) and the primers listed in Table S3. To construct the recombinant plasmids (pEAQ-CsPIF3b-GFP), the coding sequence without stop codons of CsPIF3b was cloned into the pEAQ-GFP vector [46]. The recombinant plasmids and pEAQ-GFP vectors were transfected into *Agrobacterium tumefaciens* strain EHA105 [47], which was then injected into *N. benthamiana* leaves [41]. Two days later, green fluorescent protein (GFP) signals were evaluated using a fluorescence microscope (Zeiss, Oberkochen, Germany).

To assess the transcriptional activity of recombinant plasmids pGBKT7-CsPIF3b, as well as positive (pGBKT7-53+pGADT7-T) and negative (pGBKT7) controls, were transfected into yeast cells (Y2H Gold). The yeast cells were grown on a synthetic defined (SD) medium lacking tryptophan (SD/−Trp) at 28 °C for 48 h. Then, transformants were selected on SD medium without tryptophan, histidine, and adenine (SD/−Trp/−His/−Ade). X-α-galactosidase (X-α-Gal) was used to confirm the transcriptional activation ability of CsPIF3b.

### 4.8. Dual-Luciferase Transient Transfection Analysis

For assaying the trans-activation of CsPIF3b to the promoters of genes related chlorophyll, the coding sequence of CsPIF3b was cloned into the pEAQ vector as effector and the promoters of *CsHEMA*, *CsPOR* were inserted into the pGreenII 0800-LUC vector as reporters. The constructed effector and reporter plasmids were co-transformed into tobacco leaves. LUC and REN luciferase activities were measured after 48 h of infiltration. The transcriptional ability of CsPIF3b to promoters of target genes was evaluated by the LUC/REN ratio.

## 5. Conclusions

A comprehensive analysis of CsPIF family in tea plants was carried out in the present study. Seven *CsPIF* genes were characterized and further classified into four groups, with high similar exon–intron structures and motif compositions within the same group. Furthermore, *CsPIF* genes play an important role in the regulation of chlorophyll metabolism as indicated by their expression patterns in tea leaves with different leaf colors. Subsequently, CsPIF3b was shown to act as a transcriptional activator to activate the expression of *CsHEMA* and *CsPOR* in chlorophyll metabolic pathways. These findings provide a valuable resource for better exploring the potential biological functions of *CsPIF* genes in tea plants.

## Figures and Tables

**Table 1:** The physiological and biochemical characterization of seven PIF proteins in tea plant.

Gene Name	Gene ID	CDS Length	Number of Amino Acid	Molecular Weight	Protein Isoelectric Point	Instability Index	Grand Average of Hydropathicity	Predicted Subcellular Localization
		(bp)		(KD)			(GRAVY)	
*CsPIF1*	TEA006532	1716	571	62.64	5.96	60.45	−0.546	Nucleus
*CsPIF3a*	TEA033210	1884	723	77.34	5.88	50.32	−0.554	Nucleus
*CsPIF3b*	TEA007077	2172	627	68.2	5.93	67.13	−0.634	Nucleus
*CsPIF7a*	TEA025875	1272	423	46.76	8.17	73.85	−0.703	Nucleus
*CsPIF7b*	TEA011633	1440	479	53.01	9.26	61	−0.558	Nucleus
*CsPIF8a*	TEA032260	1251	416	44.84	8.3	43.83	−0.332	Nucleus
*CsPIF8b*	TEA023842	1290	429	47.17	8.7	52.96	−0.592	Nucleus

## Data Availability

Data are contained in Supplementary Materials.

## Supplementary Materials

The following are available online at https://www.mdpi.com/article/10.3390/ijms222010949/s1, Figure S1: The similarity of amino acid sequence of PIF7 in *C. sinensis* and *T. cacao*, Table S1: The CDS sequences and amino acid sequences of *CsPIF* genes, Table S2: The details of motif compositions in CsPIFs proteins, Table S3: Summary of primers used in this study, Text S1: Sequences of PIF protein in *C. sinensis* and 13 other plant species, Text S2: Promoter sequences of structural genes in chlorophyll metabolic pathway.

## Abbreviations

PIFs	phytochrome-interacting factors
Cs	*Camellia sinensis*
ALA	5-aminolevulinic acid
APB	domain Active phytochrome B-binding domain
APA	domain Active phytochrome A-binding domain
qRT-PCR	quantitative real-time polymerase chain reaction
HEMA	glutamyl-tRNA reductase
HEME	uroporphyrinogen decarboxylase
CHLI	Mg-protoporphyrin IX chelatase subunit ChlI
POR	protochlorophyllide oxidoreductase
CAO	chlorophyll a oxygenase
SGR	STAY-GREEN
