# Supplementary material for: Identification, Molecular Characteristic, and Expression Analysis of PIFs Related to Chlorophyll Metabolism in Tea Plant (Camellia sinensis)
Source: Int J Mol Sci. 2021 Oct 11;22(20):10949. doi: 10.3390/ijms222010949 (PMC8539375; doi:10.3390/ijms222010949)

Supplementary Figure S1: Floral organ development and morphogenesis of *A. graminifolia*. A-C: Flower bud differentiation period 1; D-G: Sepal growth period 2; H-K: Pillar development period 3; L-O: Flower dyeing period 4; P-Q: Flowering period 5; A: The inflorescence; B: The inflorescence longitudinal section; C: Flower 1 (Fl 1); D, H, L: The whole flower bud; E, I, M: The longitudinal section of the flower bud; F, J, N: The pillar; G, K, O, Q: The organ anatomy; P: The flower; Se: Sepal; LSe: The lateral sepal; DSe: The dosal sepal; LPe: Lateral petal; La: Labe llum; Pi: Pillar; Br: Bract; Fl 1, Fl 2, Fl 3: Flower 1, F lower 2, Flower 3; An: Anther; Ro: Rostellum; St: Stigma; Sty: Style; Anc: Anther cap.

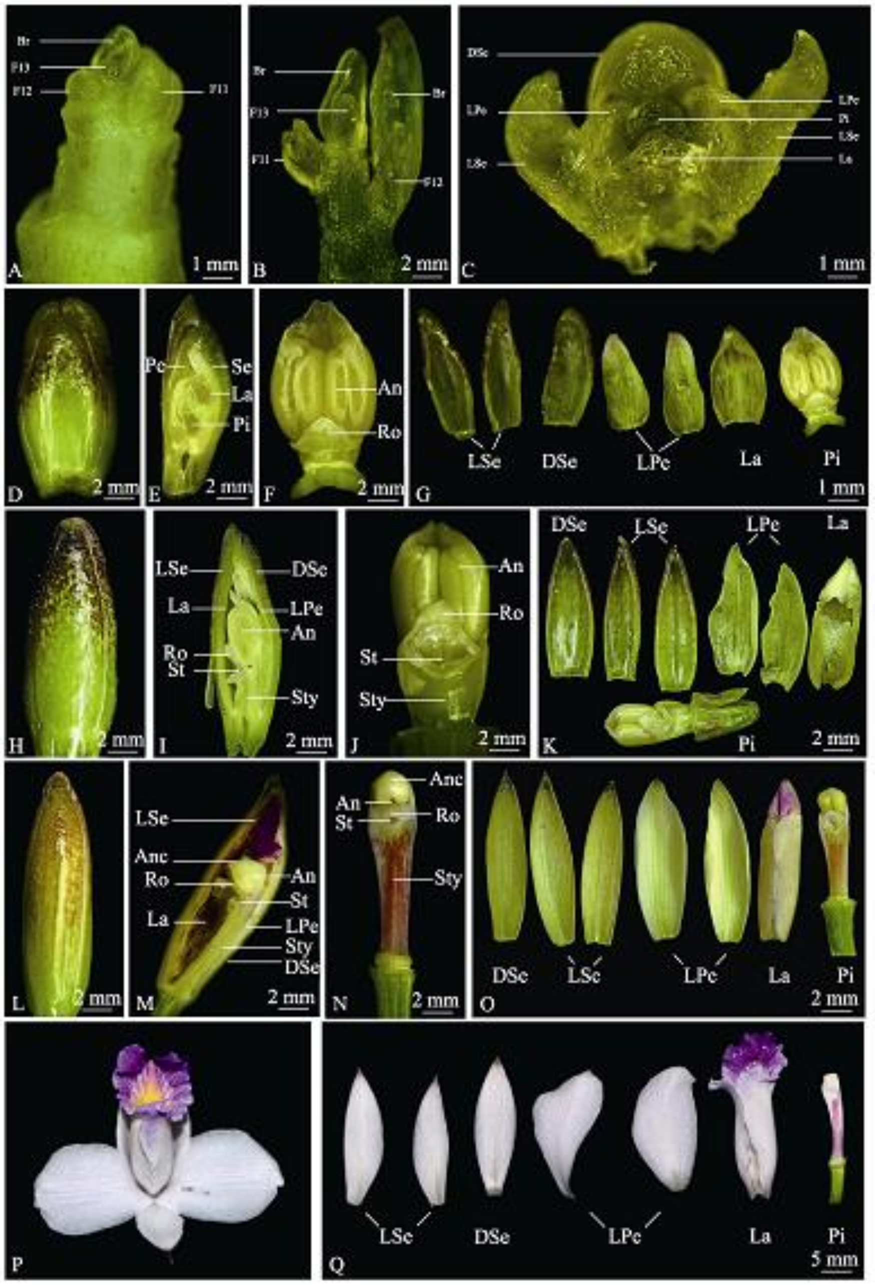

Supplement: Supplementary file 1 [file ijms-22-10949-s001.zip › Supplementary Figure.pdf]
